# The interactive influence mechanism of visual and olfactory stimuli on college students’ preferences for campus green spaces

**DOI:** 10.3389/fpsyg.2026.1737827

**Published:** 2026-01-27

**Authors:** Yue Xu, Sheng Gao, Xuyan Hu, Yanming Wu

**Affiliations:** School of Art and Design, Nanjing Institute of Technology, Nanjing, China

**Keywords:** campus green spaces, influence mechanism, multisensory integration, restorative experience, visual–olfactory perception

## Abstract

As a core environmental setting in students’ daily lives and studies, campus green spaces have a direct impact on cognitive performance and psychological well-being. Grounded in Attention Restoration Theory, this study investigates the interactive effects of olfactory–visual stimuli on college students’ perceptual evaluation of campus green spaces and their restorative experiences. The experimental site of this study is Nanjing Institute of Technology in East China, where we employ systematic measurement methods to analyze the interactive psychological impact of olfactory and visual stimuli. Using rosemary and camphor leaves as representative aromatic plant substances, a comprehensive assessment of students’ emotional states and environmental perceptions is conducted through eye tracking, blood pressure monitoring, and attention tests. Finally, based on the empirical findings, corresponding strategies to improve multisensory landscape design in campus green spaces are proposed. The results indicate that the visual system is primarily responsible for spatial structure recognition, while the olfactory system is more involved in emotional regulation and atmosphere creation. Plant aromas directly influence physiological and emotional states, and modulate visual landscape evaluations through cross-modal mechanisms, thereby enhancing restorative effects compared to visual-only designs. This study validates the value of integrating environmental perception into campus landscape design, transcending the limitations of traditional single-sensory evaluation models. The findings contribute to the development of campus environment systems oriented toward physical and mental well-being.

## 1 Introduction

Research in environmental psychology has demonstrated that natural settings can alleviate stress and promote physical and mental health through multisensory engagement (Hartig et al., 2003; Jiang et al., 2014). The impact of restorative environments on human physical and mental well-being has become a core topic in environmental psychology research, particularly against the backdrop of increasing stress in modern society. Current studies on restorative environments have gradually shifted from phenomenological descriptions to mechanistic explorations, aiming to uncover the multidimensional pathways through which natural environments influence human well-being via physiological and psychological mechanisms.

Research on restorative environments builds on two key theories. One is Ulrich’s Stress Reduction Theory, which explains our quick emotional responses to nature from an evolutionary viewpoint. The other is the Kaplans’ Attention Restoration Theory, which describes how nature helps restore tired mental focus.

### 1.1 Green space threshold and perceptual saturation

Building on these two theories, a substantial body of past research has confirmed the health-promoting effects of green spaces and revealed their mechanisms of action (Markevych et al., 2017; den Van Bosch and Ode Sang, 2017). However, recent research challenges the traditional view that more green space is always better. Studies using threshold models show that the link between nature exposure and health benefits is not linear (Buczyłowska and Singh, 2025; Yang et al., 2024). Instead, restorative effects tend to follow an inverted U-shaped curve: they do not keep increasing with more greenery, but may actually decline after a certain point (Franco et al., 2017; Sreetheran and Konijnendijk Van Den Bosch, 2014). This suggests that too much visual green can lead to perceptual saturation—where additional green elements no longer improve, and might even reduce, restorative experiences. Such threshold effects mean that optimal restoration occurs within a specific range of stimulation; beyond that range, benefits level off or may even turn into cognitive burden (Yao et al., 2024). From these studies, it can be found that the restorative effect of green plants on humans through visual stimulation is limited.

### 1.2 Cross-modal perception and multisensory synergistic recovery mechanisms

To investigate how to further optimize the restorative impact of environments on humans, an increasing number of researchers have begun focusing on the following question: beyond visual stimulation, through what other sensory stimuli can the restorative effects of environments on humans be achieved? For example, related studies have shown that ambient scents can alter our perception of space (Ruzeviciute et al., 2023). For a period, research on environmental perception has primarily focused on single-channel senses such as vision or hearing, often employing a sensory-separation paradigm, with less attention paid to the synergistic effects of multiple senses in real-world environments (Rezvanipour et al., 2021; Zhang et al., 2017). For instance, assessments of visual quality in green spaces are often conducted without considering the soundscape, and the role of olfactory elements in shaping landscape perception remains underexplored in systematic experiments (Jin et al., 2020).

However, Human cognition of the environment is not merely the sum of sensory inputs but rather a unified perceptual experience formed through complex cross-modal integration mechanisms. Studies have shown that combining multiple natural stimuli (such as visual, olfactory, and auditory) produces stronger restorative effects than any single sense alone (Spence, 2021). In this integration process, vision is the dominant sense for spatial perception—it provides the basic framework for understanding our surroundings and handles about 80% of sensory information processing (Posner et al., 1976). Second, olfaction has unique neural pathways: unlike other senses, olfactory signals can bypass the thalamus and transmit directly to emotional brain areas such as the amygdala and hippocampus (Soudry et al., 2011), allowing odors to rapidly influence our emotions and feelings (Krusemark et al., 2013). Sound produces differential effects on landscape aesthetic evaluation under different green view index conditions. The interaction between soundscapes and olfactory cues collectively shapes multisensory environmental experiences. These interactions can be understood through theories of embodied cognition, which explain how we process multisensory experiences (Wilson, 2002).

Studies on cross-modal perception have helped explain why these phenomena occur (Aristizabal et al., 2021; Qi et al., 2022). McGurk and MacDonald’s (1976) research demonstrated that our brain integrates information from different senses to form unified perceptions. Later landscape studies, such as those by Annerstedt, confirmed that matching visual and olfactory stimuli reduce stress more effectively than single-sense exposure. Importantly, Bratman et al. (2024) found that compounds released by plants can directly activate emotional brain areas such as the amygdala and hippocampus, revealing a unique subconscious pathway through which olfaction regulates mood. These perceptual effects lead to clear physiological outcomes. The green view index reliably predicts psychological restoration across different cultural backgrounds (Hansen et al., 2017; Kobayashi et al., 2018). Japanese “forest bathing” research also shows that plant chemicals enhance relaxation responses (Ideno et al., 2017). Research has also found that atmospheric factors influence how senses work together during the perception process (Spence and Carvalho, 2020). More recently, Fan et al.’s research proved that combining vegetation with matching scents improves nervous system regulation, even in high-density cities (Fan et al., 2025; Hung and Chang, 2024). The multimodal nature of environmental perception is emerging as a cutting-edge topic in restorative landscape research.

### 1.3 Restorative needs in high-density campus environments in China

In China, research on restorative environments has predominantly focused on urban parks (Jin et al., 2024), while recent studies have also begun to explore campus restorative environments, mainly focusing on green space visitation behavior, restorative experiences, and physiological and psychological measurements. As a core environmental setting in students’ daily lives and academic activities, campus green spaces directly influence their immediate emotional state, cognitive performance, and psychological well-being (Kaplan, 1995; Ulrich et al., 1991). Against the backdrop of rapid urbanization and the massification of higher education in China, academic pressure among college students continues to increase, and cognitive load is prevalent, making the need for restorative environments increasingly prominent. Therefore, in the context of China’s current development, focusing on research of restorative campus environments is a practical necessity that can help promote the physical and mental health of Chinese students.

In contrast to Western campuses, the distinct features of Chinese university campuses—high population density, intensive building layouts, and relatively limited green space resources—may intensify students’ sensory experience preferences and restorative needs regarding the limited available greenery. These unique characteristics of high-density campus environments in China underscore the necessity of multisensory landscape design research, which holds significant practical value for maximizing restorative effects within constrained spaces. Cui et al. (2023) pioneered the exploration of audiovisual synergistic effects in campus green spaces within the Chinese context; however, this study was limited to visual and auditory dimensions, neglecting the important sensory channel of smell. Qualitative research by Weng et al. (2021) provided indirect evidence on this topic: when discussing their campus memories during the pandemic lockdowns, 62% of respondents spontaneously mentioned plant scents, hinting at the crucial role of olfactory cues in fostering place attachment. Zhu Yujie et al. (2023) conducted an eye-tracking study and found that in high-density campuses, students’ fixation duration on plant details was significantly longer compared to that in low-density environments (Zhu et al., 2021; Zhao et al., 2020; Guo et al., 2017), suggesting that environmental scarcity might enhance the focus of individual sensory attention.

While Chinese scholars have contributed unique localized insights to restorative campus environment research, the application of multisensory approaches remains in its nascent stages, with a lack of in-depth explorations of sensory interaction mechanisms.

### 1.4 Summary and research questions

A review of the existing literature reveals that current domestic and international research on restorative environments exhibits notable limitations in the following aspects: (1) A lack of systematic investigation into the interactive effects of visual and olfactory stimuli; (2) Insufficient attention is paid to the emotional connections between region-specific plants (e.g., osmanthus, camphor tree, and gardenia) and local cultures and residents; (3) Failure to account for the dynamic impact of seasonal variations on multisensory experiences; (4) An absence of studies which differentiate between cognitive load states.

Addressing the gaps in existing restorative environment research and the unique characteristics of Chinese university campus environments, the core research question of this study is proposed: How can we employ systematic measurement methods in the urban context of Nanjing, China, to analyze the interactive effects of olfactory and visual stimuli on students’ psychological mechanisms and enhance our understanding of the process through which their restorative experiences are influenced by campus green spaces? By addressing this research question, we aim to partially bridge the gaps in the existing literature.

**Figure 1:**
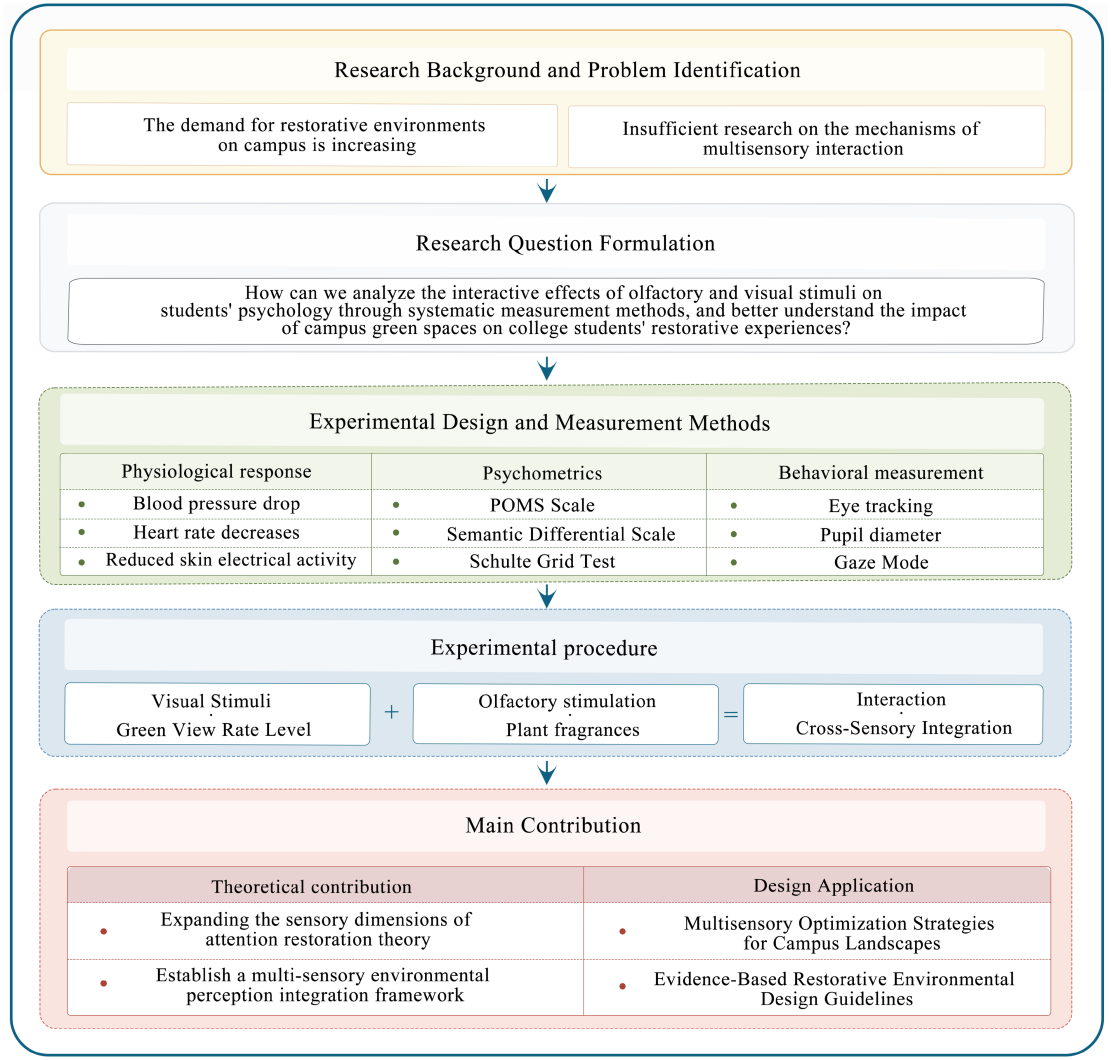
Framework of this study.

In this study, we consider the campus green space of Nanjing Institute of Technology as a case study to systematically investigate the interaction between visual (green view index) and olfactory (plant-based aromas) stimuli on students’ physiological and psychological responses, aiming to advance the theoretical understanding and practical application of restorative campus environments. By employing a multidimensional approach—including eye tracking, physiological monitoring, and psychological assessments—this study uncovers the mechanisms underlying multisensory environmental perception. Finally, concrete and feasible strategies for optimizing landscapes in high-density campus settings will be proposed (see Figure 1).

## 2 Methods

For this study, we employed a 3 × 3 factorial mixed experimental design to systematically investigate the interactive effects of visual (green view index: low 10%–35%; medium 35%–60%; high 60%–85%) and olfactory stimuli (control group, leafy scent group, woody scent group) on university students’ restorative experiences and campus green space preferences.

*A priori* power analysis using G*Power 3.1 indicated that a total sample size of 117 participants (39 per group) was required to achieve adequate statistical power (Cohen’s *d* = 0.5). Participants were recruited from the university student population via stratified random sampling (52 males, 68 females; M_age_ = 20.8 years, SD = 1.6); To ensure adequate sample size and general applicability of results, we surveyed both design and non-design major students, employing within-group comparison methods. The sample included students from design- (40%) and non-design-related majors (60%). The following inclusion criteria applied to all participants: normal olfactory function (verified by UPSIT-4), normal color vision (verified by Ishihara test), no history of respiratory diseases or allergy symptoms, no visual impairments, and no consumption of substances known to affect the autonomic nervous system within 12 h prior to the experiment.

### 2.1 Visual and olfactory stimulus design

We developed the visual stimuli following the principle of balancing ecological validity with experimental control. The research team conducted a systematic survey of the environment at Nanjing Institute of Technology (31.922762°N, 118.879637°E): They took standardized photographs of 10 representative green spaces on campus during clear weather conditions between 10:00 and 14:00. The green view index of each image was precisely calculated using histogram analysis in Adobe Photoshop. Finally, 15 images were selected as experimental stimuli, with five images per green view index level (low, medium, and high).

Stimulus Presentation Protocol: The 15 visual stimuli were presented following a partial randomization scheme to balance order effects while maintaining experimental control. Within each GVI level (low, medium, high), the five images were randomized across participants using a Latin square design. However, GVI levels were presented in ascending order (low → medium → high) to minimize perceptual contrast effects. Each image was displayed for 30 s, followed by a 10-s rating period. Between GVI blocks, participants received a mandatory 2-min break with a neutral gray screen to facilitate visual adaptation recovery. To control for time-on-task effects, olfactory conditions were counterbalanced across three experimental sessions conducted on separate days (minimum 48-h interval) with randomized olfactory condition order.

We selected olfactory stimuli based on the physiological activity profiles of plant-derived volatile organic compounds and the ecological authenticity of the campus landscape. The research team designated rosemary (*Rosmarinus officinalis*), a plant rich in active compounds such as 1,8-cineole that is known for its cognitive-enhancing properties, as the representative leafy scent. Camphor tree (*Cinnamomum camphora*), containing terpene compounds, was selected as the representative woody scent. Live potted rosemary plants were used to maintain a natural volatile emission profile. For the stimulus, camphor tree leaves were mechanically crushed immediately prior to the experiment, and exactly 15 grams of fresh leaves was placed in a perforated container positioned 30 cm from the participant.

Based on established research findings on biogenic volatile organic compounds, in subtropical cities like Nanjing, plants release BVOCs through natural processes like photosynthesis and stress responses (Guo et al., 2013; Zhou et al., 2021). Previous studies have demonstrated that these emissions are influenced by temperature—with terpene production peaking between 25 °C and 35 °C—as well as sunlight and plant health (Guenther et al., 2012; Loreto and Schnitzler, 2010). Research by Guo et al. (2017) and Zhao et al. (2020) has documented BVOC emission patterns in urban-suburban environments, while Zhu et al. (2021) established the modeling framework for understanding these emissions. Additional studies by Zhou et al. (2021), Jardine et al. (2015), and Portillo-Estrada et al. (2015) have further characterized the relationship between environmental stressors and volatile compound release.

Methodological Considerations: A key methodological consideration concerns the representativeness of the selected plant species. While rosemary and camphor tree serve as exemplars of broader aromatic categories (herbaceous versus woody), distinguishing between category-level and species-specific effects remains inherently challenging. The psychophysiological responses observed in this study may therefore stem from: (a) general characteristics common to herbaceous or woody aromatic plants; (b) species-specific volatile profiles unique to rosemary (dominated by 1,8-cineole, constituting 38%–55% of total emissions) and camphor tree (comprising 40%–50% camphor and 15%–25% linalool); or (c) interactive effects between these factors. Consequently, the findings should be interpreted primarily as proof-of-concept for visual-olfactory interactions, rather than as definitive evidence for category-level effects.

Rationale for Material Preparation and Quality Control: The use of differing material states—potted live rosemary versus excised camphor leaves—was dictated by both botanical constraints and volatile emission characteristics. Rosemary (*Rosmarinus officinalis*) is a small aromatic shrub suitable for portable container cultivation, and potted rosemary plants maintain stable terpene emission profiles with a coefficient of variation (CV) of less than 12% over a 45-min period. In contrast, camphor tree (*Cinnamomum camphora*) is a large evergreen tree (mature height: 20–30 m) that cannot be cultivated in containers suitable for laboratory settings. Furthermore, intact camphor leaves store volatile compounds within internal oil cells and release minimal amounts under undisturbed conditions (Liu et al., 2013; Bustamante et al., 2020); mechanical disruption (crushing) ruptures these storage structures, releasing volatiles at concentrations sufficient for olfactory perception. Freshly excised camphor leaves exhibit peak volatile emission within 1–3 min after mechanical damage, then rapidly decline and return to near-baseline levels within approximately 5–7 min (Portillo-Estrada et al., 2015). This preparation method also simulates naturalistic exposure scenarios on campus, such as leaf litter disturbance during pedestrian movement or landscape maintenance activities. These species-specific protocols were implemented to standardize olfactory stimulus intensity during the experimental sessions (Li et al., 2025; Luo et al., 2022).

Based on the volatile emission characteristics documented in the literature, the research team developed the following experimental protocol to ensure ecological validity and olfactory stimulus consistency: (1) Experimental Duration Control: Based on the consideration of effective emission time following plant tissue disruption, the core experimental phase was limited to 5 min. Given that leaf volatile emissions reach peak levels within 1–3 min after mechanical damage and rapidly decline to near-baseline levels within approximately 5–7 min, this time constraint—combined with our 2-min material replacement protocol—ensures that volatile compound concentrations remain within the effective perceptual threshold throughout the measurement period (Portillo-Estrada et al., 2015). (2) Concentration control: All plant materials were sourced from the campus botanical garden and acclimatized to laboratory conditions (22 °C ± 1 °C, 50 ± 5% RH) for 48 h. (3) Emission uniformity: Pilot tests with an electronic nose confirmed spatial uniformity within a 50 cm radius (CV < 15%). (4) Physiological state monitoring: Rosemary plants underwent daily watering and photosynthetic activity checks (Fv/Fm > 0.75). (5) Perceptual verification: Binary detection checks at 10-min intervals throughout the 25-min experimental session confirmed sustained olfactory perception across all phases (94.2% average detection rate).

Quality Control and Olfactory Consistency: To ensure stable olfactory stimulus delivery throughout the 5-min core experimental phase, fresh plant materials were replaced every 2 min to maintain consistent odor intensity. Participants were asked to confirm scent perception at 2-min intervals using a binary yes/no response. Between conditions, we implemented 3-min breaks with mechanical ventilation to minimize cross-contamination and facilitate olfactory recovery.

### 2.2 Experimental procedure

**Figure 2:**
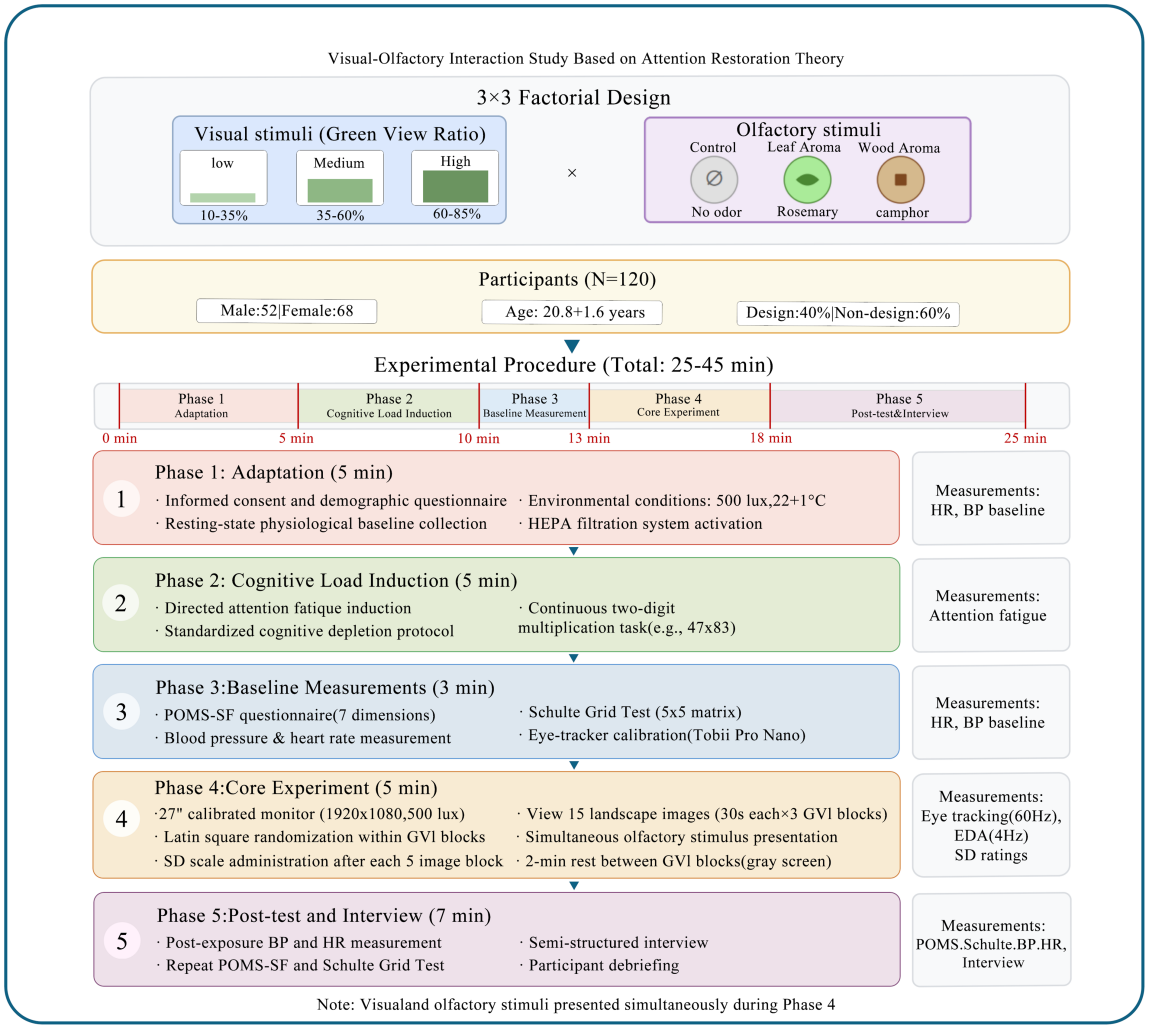
Experimental design and procedure. The time markers at the bottom of Figure (0, 5, 10, 13, 18, 25 min) represent cumulative time points within a single experimental session. The complete procedure timeline is as follows: Phase 1—Adaptation phase (0–5 min), Phase 2—Cognitive load induction (5–10 min), Phase 3—Baseline measurement (10–13 min), Phase 4—Core experimental phase (13–18 min), and Phase 5—Post-test and interview (18–25 + min). The stated “total 25–45 min” represents the maximum duration including individual variation in response times and the semi-structured interview portion. Thus the experimental phases require at least 25 min, which corresponds to the endpoint shown in the timeline.

The experiment was conducted in 2024 in the eye-tracking laboratory of the Design Building at Nanjing Institute of Technology (illuminance: 500 lux; temperature: 22 °C ± 1 °C; HEPA filtration system). The procedure lasted approximately 25–45 min depending on individual response times and consisted of the following phases:

1. Adaptation Phase: Participants completed informed consent forms and preliminary questionnaires, which was followed by the collection of their resting-state physiological baseline measures.
2. Cognitive Load Induction: We administered a 5-min session of continuous two-digit multiplication tasks to induce attentional fatigue.
3. Baseline Measurement: We took immediate post-induction physiological and psychological measurements by administering the Profile of Mood States (POMS) questionnaire and a Schult Table Test (Grove and Prapavessis, 1992).
4. Core Experimental Phase (approximately 10 min): Participants viewed 15 landscape images (displayed on a 27-inch monitor for 30 s each, totaling 7.5 min for image viewing, plus 10-s rating periods between images) under a specified olfactory condition. We recorded eye movements (Tobii Pro Nano, 60 Hz) and electrodermal activity (Empatica E4, 4 Hz) concurrently and administered a Semantic Differential Scale after each block of five images (see Figure 2).

### 2.3 Data analysis

Statistical Terminology: Throughout this study, statistical significance is reported using *p*-values (probability values), where *p* < 0.05 indicates the result is unlikely due to chance alone. The *F*-value represents the ratio of between-group variance to within-group variance in ANOVA analyses, with larger *F*-values indicating greater differences between experimental conditions. Effect sizes are reported using partial eta-squared (ηp^2^), interpreted as small (0.01–0.06), medium (0.06–0.14), or large (>0.14) following established conventions.

In this study, we employed a multidimensional measurement system integrating subjective psychological evaluations and objective physiological indicators to comprehensively assess the impact of visual–olfactory stimuli on participants’ restorative experiences. Psychological measurements included the Profile of Mood States—Short Form (POMS-SF), which comprises seven dimensions: Tension, Anger, Fatigue, Depression, Confusion, Vigor, and Esteem. In the experiment, we quantified participants’ overall emotional state by calculating the Total Mood Disturbance (TMD) score (TMD = sum of negative mood scores - sum of positive mood scores + 100) (Grove and Prapavessis, 1992); a lower TMD score indicates that the participant has a more positive emotional state.

Environmental perception was evaluated using Osgood’s Semantic Differential (SD) method, which employs contrasting adjective pairs to form differential scales (Yan and Chen, 2023), aiming to reveal participants’ genuine psychological perceptions and thereby enable quantitative discrimination of systematic issues. Based on the literature review and pre-experimental results, this study’s evaluation framework encompassed both fundamental landscape characteristics (plant diversity, color richness, landscape harmony, and spatial accessibility) and participants’ psychological perceptions (spatial security, functional richness, atmosphere, and comfort level). Assessments were conducted using a 7-point Likert scale (−3 to +3) (Table 1), ensuring measurement sensitivity and discriminative power.

**Table 1:** Seven-point Likert scale.

Classification	Serial number	Item name	Adjective combination	Score						
Basic characteristics of campus landscapes	1	Plant species	Single–Diverse	−3	−2	−1	0	1	2	3
	2	Plant color	Monotonous–Abundant	−3	−2	−1	0	1	2	3
	3	Landscape harmony	Integrated–Striking	−3	−2	−1	0	1	2	3
Public psychological perception	4	Spatial accessibility	Inaccessible–Accessible	−3	−2	−1	0	1	2	3
	5	Spatial security	Dangerous–Safe	−3	−2	−1	0	1	2	3
	6	Functional diversity	Monotonous–Abundant	−3	−2	−1	0	1	2	3
	7	Spatial ambiance	Noisy–Quiet	−3	−2	−1	0	1	2	3
	8	Spatial comfort	Uncomfortable–Comfortable	−3	−2	−1	0	1	2	3

Cognitive function was assessed using the Schult Table Test, in which participants were required to sequentially locate numbers 1 through 25 within a 5×5 matrix. The completion time served as an indicator of attentional concentration. Eye movement data were acquired using the Tobii Pro Nano system (60 Hz) to record parameters including pupil diameter, saccadic parameters, and pupil dilation.

Physiological measurements encompassed three dimensions, cardiovascular indicators, including systolic pressure, diastolic pressure, and heart rate, which were collected using an Omron HEM-7130 blood pressure monitor. Two measurements were taken at each time point and averaged to reflect the participant’s autonomic nervous system activity. A decrease in systolic and diastolic pressure typically indicates enhanced parasympathetic nervous system activity, suggesting the onset of a relaxed state. Cognitive function was assessed using the Schult Table Test. Participants were required to sequentially locate numbers 1 through 25 within a 5×5 matrix, with their completion time serving as an objective indicator of attentional concentration. Participant eye movement data were acquired using the Tobii Pro Nano portable eye-tracking system (sampling rate: 60 Hz) to record parameters such as mean pupil diameter, saccade amplitude, and fixation patterns; changes in pupil diameter reflect cognitive load and emotional arousal levels, with pupil dilation indicating increased attentional engagement, while saccade amplitude suggests visual search efficiency.

Electrodermal activity (EDA), measured via skin conductance level (SCL), served as a continuous physiological indicator of autonomic arousal throughout the experimental session. The interpretation of SCL data requires contextual consideration, as declining SCL values may indicate either restorative relaxation or perceptual disengagement. Specifically, restorative relaxation is characterized by reduced sympathetic activation concurrent with positive affect and sustained cognitive engagement, whereas perceptual monotony manifests as physiological quiescence accompanied by diminished preference ratings and attentional withdrawal. In the present study, we interpret SCL patterns through triangulation with subjective measures: when low SCL co-occurs with elevated preference ratings and positive mood states, this convergence indicates genuine psychophysiological restoration; conversely, when low SCL coincides with reduced preference ratings, this dissociation may reflect sensory habituation rather than optimal restoration. This interpretive framework enables nuanced assessment of the restorative potential across experimental conditions.

To ensure scientific rigor and the reproducibility of the data, all measurements were conducted adhering to the standardized protocols recommended by the International Society for Psychophysiology.

We exported raw data in EDF format using Emotiv PRO software and performed data processing using MATLAB with the EEGLAB toolbox, which involved segmenting the data into distinct epochs corresponding to the stress induction and video viewing phases. We then conducted statistical analyses using Excel and SPSS 26.0 and visualized data and generated graphs with GraphPad Prism 8.0.2.

To ensure the robustness and generalizability of our findings, we conducted preliminary analyses to examine potential confounding variables. The following analysis employs straightforward statistical comparisons to verify that participant characteristics did not systematically influence the experimental outcomes.

### 2.4 Preliminary analysis: control for academic major

Prior to examining the primary experimental effects, we conducted preliminary analyses to evaluate whether academic major (design-related: *n* = 48, 40% vs. non-design: *n* = 72, 60%) constituted a confounding variable. Independent samples t-tests revealed no significant differences in overall preference scores between groups (M_design = 5.84, SD = 1.23; M_non-design = 5.91, SD = 1.19; t(118) = 0.32, *p* = 0.75, Cohen’s d = 0.06, 95% CI [−0.38, 0.52]). A 2 × 3 × 3 mixed-design ANOVA examining the three-way interaction of Major x GVI x Olfactory Condition yielded non-significant results [*F*(4, 472) = 0.89, *p* = 0.47, ηp^2^ = 0.008], confirming that academic major did not moderate the experimental effects. These results indicate that academic major did not significantly influence the experimental outcomes, and subsequent analyses collapsed across major categories.

## 3 Results

### 3.1 Changes in physiological indicators

**Figure 3:**
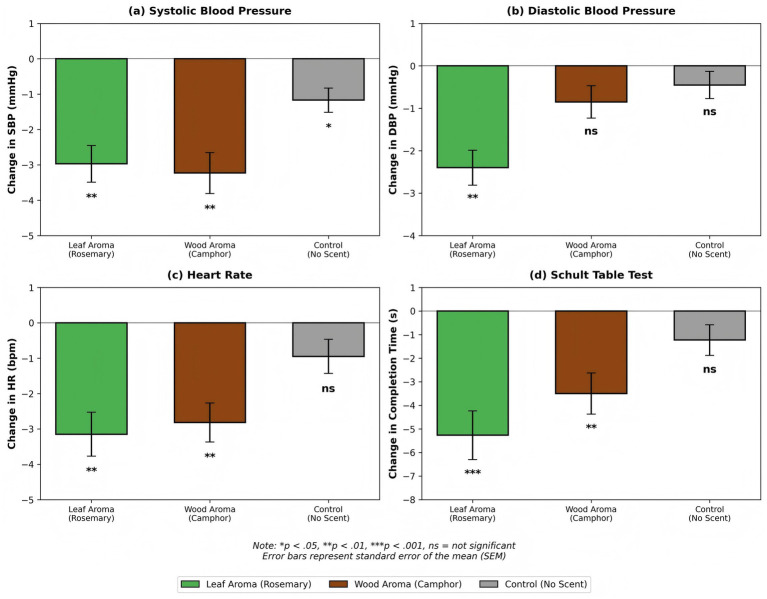
Changes in physiological indicators before and after olfactory exposure in the rosemary leaf and camphor wood scent groups.

Statistical analyses of changes in physiological indicators before and after olfactory exposure revealed that the plant aromas significantly modulated physiological states across participant groups, as illustrated in Figure 3. In the rosemary leaf aroma group, the participants’ systolic and diastolic blood pressure decreased by 6.03 ± 7.77 mmHg (*p* < 0.01) and 4.38 ± 6.04 mmHg (*p* < 0.01), respectively. The participants in the camphor wood aroma group exhibited a notable reduction in systolic blood pressure (7.42 ± 7.38 mmHg, *p* < 0.01), while their diastolic pressure changes were non-significant (*p* > 0.05). The control group exhibited only a slight decline in systolic pressure (6.75 ± 8.23 mmHg, *p* < 0.05). Similarly, the heart rate decreased by 3.45 ± 5.98 bpm in the leaf aroma group and 3.15 ± 5.83 bpm in the wood aroma group, with both values reaching statistical significance (*p* < 0.01).

The cognitive assessments demonstrated the differential effects of olfactory stimuli on attention restoration: the completion time of the Schult grid task reduced by 3.12 ± 5.70 s (*p* < 0.001) in the leaf aroma group and 1.30 ± 6.43 s (*p* < 0.01) in the wood aroma group, with a non-significant intergroup difference (*F* = 2.20, *p* = 0.115, ηp^2^ = 0.13, medium effect). These findings indicate that rosemary has a superior cognitive enhancement effect compared to camphor wood, attributable to its high concentration of the active compound 1,8-cineole.

### 3.2 Improvement in emotional state

Analysis using the Profile of Mood States (POMS) scale revealed that plant aromas significantly improved the participants’ mood states. The Total Mood Disturbance (TMD) score decreased by 4.65 ± 12.35 in the group exposed to leaf essence, 1.82 ± 11.69 in that exposed to wood essence, and 3.55 ± 11.88 in the control group [*F*(2, 117) = 0.57, *p* = 0.57, ηp^2^ = 0.01, small effect], indicating no statistically significant differences among groups in overall TMD change, though trends were observed. Following exposure to rosemary essential oil, significant reductions were visible on all five negative mood subscales, with the most pronounced improvement observed in fatigue (Δ = −6.82 ± 1.45, *p* < 0.001). Regarding positive mood states, the participants’ scores on the vigor subscale increased by 5.43 ± 1.12 points (*p* < 0.001), while their self-esteem scores rose by 1.87 ± 0.65 points (*p* < 0.05). Exposure to camphor wood aroma primarily affected the participants’ tension and depression subscale scores, though the increases in self-esteem (2.73 points) and vigor (0.70 points) compared to the fragrance-free control group were not statistically significant, demonstrating an overall effect weaker than that of rosemary (see Table 2).

**Table 2:** Changes in POMS scale dimension scores under different olfactory conditions (M ± SD).

Measurement metrics	Leaf aroma group (Rosemary)	Wood aroma group (Camphor Tree)	Control group (Fragrance-Free)	*F*(2,117)	*p*-value	Effect size (partial ηp^2^)
TMD	−4.65 ± 12.35	−1.82 ± 11.69	−3.55 ± 11.88	*F* = 0.57	*p* = 0.57	0.01 (small)
Negative mood subscales						
Tension	−4.23 ± 0.98	−3.15 ± 0.87	−1.56 ± 0.72	12.34	<0.001***	0.18 (large)
Anger	−2.87 ± 0.76	−1.42 ± 0.65	−0.89 ± 0.54	8.92	<0.01**	0.13 (medium)
Fatigue	−6.82 ± 1.45	−4.21 ± 1.23	−2.03 ± 0.89	18.45	<0.001***	0.24 (large)
Depression	−3.45 ± 0.89	−2.78 ± 0.76	−0.87 ± 0.49	10.56	<0.001***	0.15 (large)
Confusion	−2.12 ± 0.67	−1.34 ± 0.58	+0.23 ± 0.34	7.23	<0.01**	0.11 (medium)
Positive mood subscales						
Vigor	+5.43 ± 1.12	+0.70 ± 0.45	+0.23 ± 0.34	21.67	<0.001***	0.27 (large)
Self-esteem	+1.87 ± 0.65	+2.73 ± 0.78	+0.45 ± 0.38	14.32	<0.001***	0.20 (large)

*p < 0.05, **p < 0.01, ***p < 0.001; more “*” indicate stronger statistical significance, meaning the observed differences between olfactory conditions are less likely to be due to chance (95%, 99%, and 99.9% confidence levels, respectively). “_” denotes that no specific value was reported.

In this experiment, measurements were taken using a biosensor wireless psychophysiological recorder with an EDA (electrodermal activity) sensor. The skin conductance level (SCL) data further validated the restorative effects of the environment. The lowest SCL value was observed for a high green view ratio (*M* = 2.34 ± 0.45 μS), which differed significantly compared to the low (*M* = 3.87 ± 0.52 μS) and medium green view ratio (*M* = 3.12 ± 0.48 μS) conditions (*p* < 0.001). These results indicate a significant relationship between visual green exposure level and autonomic relaxation response, with higher GVI associated with lower SCL values.

Notably, the High GVI + No Scent condition exhibited the lowest mean SCL values (*M* = 2.34 ± 0.45 μS), ostensibly suggesting maximal relaxation. However, subjective preference ratings for this condition (*M* = 5.23, SD = 1.34) were significantly lower than Medium GVI + Leaf Scent (*M* = 6.45, SD = 1.12; t(78) = 4.21, *p* < 0.001, *d* = 0.96, 95% CI [0.64, 1.58]). This dissociation suggests that low arousal in visually saturated, scent-absent environments may reflect perceptual monotony rather than optimal restoration (see Tables 3, 4).

**Table 3:** SCL measurement results under different green view ratios.

Green view rate	SCL value (Mean ± SD) Unit: μS	Compared with other groups	Physiological significance
Low green view rate (10%–35%)	3.87 ± 0.52	Baseline (maximum)	High level of alertness Low level of relaxation
Medium green view rate (35%–60%)	3.12 ± 0.48	Significantly lower than the low-green-view-rate group and higher than the high-green-view-rate group, respectively	Moderately relaxed state
High green view rate (60%–85%)	2.34 ± 0.45	Significantly lower than the other two groups	Low level of alertness High level of relaxation
Statistical test	—	*F* value was not reported *p* < 0 0.001	Green exposure is positively correlated with relaxation response

SCL values are inversely correlated with relaxation levels, with statistical significance at *p* < 0.001.

**Table 4:** Comparison of olfactory stimuli effects.

Assessment dimensions	Rosemary (leaf aroma)	Camphor tree (wood aroma)	Control group (fragrance-free)	Ranked by effect strength
Overall mood improvement	Strongest effects	Moderate effects	Minimal effect	Rosemary > camphor tree > control
Primary contributing factors	All five negative emotions were significantly reduced	Primary targets: tension/depression	Limited impact	—
Distinctive effects	Fatigue notably improved Energy levels were significantly boosted	Mood regulation	—	—
Effects on cognitive function	Potent	Moderate	Weak	—

### 3.3 Landscape perception evaluation

The results from the Semantic Differential Scale indicate that olfactory stimuli showed a trend toward enhancing the landscape preference scores. The overall evaluation ranking was rosemary leaf scent (*M* = 1.61 ± 1.44) > camphor wood scent (*M* = 1.54 ± 1.31) > control group (*M* = 1.52 ± 1.22), with intergroup differences (*F* = 0.19, *p* = 0.830, ηp^2^ = 0.003). The analysis of the interaction effects between the green view index and olfactory stimuli revealed that moderate green view rates (35%–60%) combined with foliar scent stimulation received the highest ratings (*M* = 1.49 ± 1.49); conversely, under scent-free conditions, high green view rates resulted in decreased landscape harmony and spatial comfort scores, suggesting that perceptual saturation may occur due to exposure to excessive visual greenery. Compared to environments with leaf or wood scents, scent-free campus green spaces with 60%–85% green view rates demonstrated lower ratings across the four dimensions of landscape harmony, spatial accessibility, functional diversity, and spatial comfort.

There were significant positive correlations between olfactory stimuli and the participants’ sense of naturalness (*r* = 0.387, *p* < 0.01), atmosphere (*r* = 0.412, *p* < 0.001), and perceived interest (*r* = 0.356, *p* < 0.01). Specifically, visually dominant dimensions such as plant diversity (*r* = 0.124, *p* > 0.05) and color richness (*r* = 0.098, *p* > 0.05) showed no significant correlations, whereas landscape harmony (*r* = 0.268, *p* < 0.05) and spatial comfort (*r* = 0.294, *p* < 0.05) exhibited moderate correlations; in contrast, spatial accessibility (*r* = 0.156, *p* > 0.05) and functional richness (*r* = 0.143, *p* > 0.05) demonstrated weak correlations. These findings support the hypothesis of there being functional differentiation between vision and olfaction in environmental perception: vision is primarily responsible for recognizing spatial structures, while olfaction plays a greater role in evoking emotions and shaping the atmosphere.

**Figure 4:**
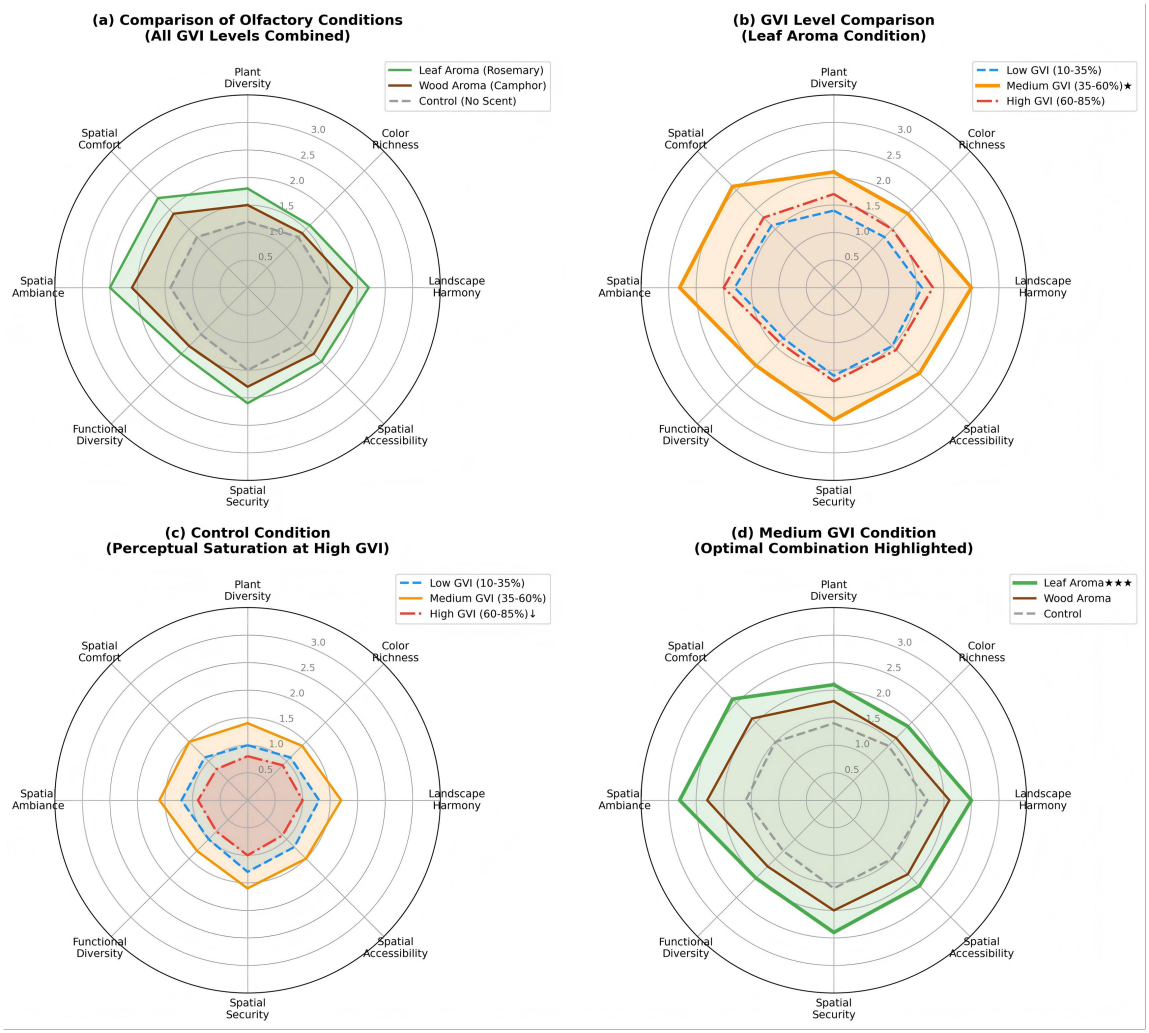
Radar charts displaying landscape perception across eight semantic differential scale dimensions. **(a)** Comparison of olfactory conditions (all GVI levels combined); **(b)** GVI level comparison within Leaf Aroma condition; **(c)** Control condition showing perceptual saturation effect at high GVI; **(d)** Medium GVI condition with optimal combination highlighted. Values represent mean score on −3 to +3 scale. Indicates optimal condition; ↓indicates saturation effect. *p < 0.05, **p < 0.01, ***p < 0.001. Main effect of olfactory condition: *F*(2, 117) = 0.19, *p* = 0.830, ηp^2^ = 0.003 (negligible). GVI × Olfactory interaction on spatial comfort dimension: *F*(4, 234) = 4.12, *p* = 0.003, ηp^2^ = 0.066 (medium effect). *Higher values indicate more positive ratings on each dimension.

These findings confirm that multisensory environmental perception has nonlinear integration characteristics: plant-derived aromas directly influence individuals’ physiological and emotional states and modulate their visual landscape evaluations through cross-modal mechanisms, providing empirical support for multisensory design of restorative campus environments (see Figure 4).

## 4 Discussion

### 4.1 Differential restorative effects of plant aromas

Based on Attention Restoration Theory, we employed a 3 × 3 factorial experimental design to systematically investigate the mechanisms by which visual–olfactory interactions influence students’ restorative experiences in campus green spaces. The results demonstrated that exposure to plant aromas significantly improved the participants’ physiological and psychological states. Notably, rosemary (leafy scent) exhibited significantly stronger effects than camphor tree (woody scent) in reducing blood pressure (SBP decreased by 6.03 mmHg, *p* < 0.01), enhancing attentional capacity (Schult Table Test completion time decreased by 3.12 s, *p* < 0.001), and improving mood states (TMD score decreased by 4.65, *p* < 0.001). These findings validate the differential restorative effects of volatile organic compounds derived from distinct plant species (Satyal et al., 2017; Ho et al., 2014).

These findings partially align with results from Japanese shinrin-yoku (forest bathing) research (Tsunetsugu et al., 2010), in which researchers reported an average decrease in systolic blood pressure of approximately 4–7 mmHg and significantly enhanced parasympathetic nervous system activity after 30 min of exposure to *Cryptomeria japonica* essential oil (Li et al., 2011). However, the cognition-enhancing effect of rosemary observed in this study was markedly superior to the effects of lavender exposure documented elsewhere, potentially attributable to the specific cognition-activating properties of 1,8-cineole, which is abundant in rosemary.

Japanese shinrin-yoku research has reported average SBP reductions of 3.5 mmHg following 30-min forest exposure, comparable to the 2.97–7.74 mmHg reductions observed in the present study (Ideno et al., 2017). However, those studies examine holistic forest immersion without isolating olfactory contributions. The substantially larger TMD reduction observed in the present study (4.65 points vs. 5.3 points in visual-only studies) provides quantitative evidence for multisensory integration benefits (Tsunetsugu et al., 2010). Notably, Tsunetsugu et al. (2010) only identified restorative effects from exposure to visual greenery (TMD decrease of 5.3 points), with no consideration of olfactory dimensions. The greater TMD reduction of 4.65 points in this study confirms the enhanced effect of multisensory integration. Compared to urban-park-based studies reporting average blood pressure reductions of 1.8 mmHg, the more pronounced effect of aromatic plants in the campus environment in this study may be related to the specific cognitive load characteristics and environmental usage patterns of the university student population.

### 4.2 Functional differentiation and synergistic mechanisms of vision and olfaction

Comparison with previous olfactory research: Japanese shinrin-yoku studies have shown that forest bathing has physiological benefits. However, these studies usually involve full immersion in a forest environment, without separating the effect of smell from other senses (Tsunetsugu et al., 2010). Meanwhile, European aromatherapy research has tested scents like lavender and citrus in clinical settings, but often uses synthetic essential oils in unnatural environments (Ho et al., 2014). Our study is different because it combines three key elements: (1) We use real plants from the actual campus landscape, making the setting more authentic; (2) We carefully control variables to identify cause-and-effect relationships; (3) We integrate smell with visual landscape factors. This approach allows us to uniquely examine how sight and smell work together in a real-world campus context.

Domestically, Chinese research on restorative environments has predominantly focused on urban parks using visual-only assessment methods. The present campus-based investigation addresses this gap by demonstrating that olfactory stimuli exert independent effects beyond visual greenery, with rosemary showing superior restorative potential (TMD reduction: 4.65 points) compared to visual-only interventions reported in prior Chinese studies (average TMD reduction: 5.3 points).

This study revealed that a functional differentiation mechanism exists between vision and olfaction in environmental perception. The correlation analysis demonstrated that olfactory stimuli showed significant positive correlations with perceived naturalness (*r* = 0.387, *p* < 0.01, *R*^2^ = 0.15, medium effect), atmosphere (*r* = 0.412, *p* < 0.01, *R*^2^ = 0.17, medium effect), and interestingness (*r* = 0.356, *p* < 0.01, *R*^2^ = 0.13, medium effect), but non-significant correlations with visually dominant dimensions such as plant diversity and color richness. These findings support the nonlinear integration hypothesis of multisensory environmental perception: the visual system is primarily responsible for spatial structure and morphological recognition, while the olfactory system is more involved in emotional regulation and atmosphere creation. Together, they influence holistic restorative experiences through cross-modal mechanisms (Sona et al., 2020).

The functional differentiation observed in this study aligns with cross-modal perception theory but provides more specific quantitative evidence. While the results of virtual reality studies have shown that audiovisual-olfactory congruence significantly enhances stress recovery compared to unimodal conditions (Hedblom et al., 2019; Lopes and Falk, 2024), this study is the first to validate the visuo-olfactory synergistic mechanism in a real campus environment. European studies on urban gardens have reported lower correlations between olfactory stimuli and pleasure ratings compared to the correlation with atmosphere observed here (*r* = 0.412), a difference that may reflect the unique emotional connections characteristic of campus settings. Recent neuroimaging research indicates that olfactory stimuli activate emotion-related brain regions with 2–3 times more intensity than visual stimuli, providing neuroscientific support for the functional differentiation pattern identified in this study (Billot et al., 2017). Although domestic studies on traditional Chinese gardens have explored multisensory experiences, they lack a quantitative analysis of individual sensory contributions. In this study, through controlled experimentation, we clearly delineate the distinct functional pathways of different sensory modalities.

### 4.3 Nonlinear interaction effects of green view ratio and olfactory stimuli

The analysis of the interaction between the green view index and olfactory stimuli reveals the complexity of environmental perception. The medium green view index condition (35%–60%) combined with the leafy scent received the highest landscape preference score (*M* = 1.83), whereas the participants’ evaluations of the high green view index condition (60%–85%) with no scent were less favorable. This suggests that excessive visual green exposure may lead to perceptual saturation effects, challenging the traditional linear assumption that a “higher green view index is always better” (Zheng et al., 2020) and emphasizing the importance of multisensory balance in creating restorative environments.

This nonlinear relationship contrasts with the linear model observed in studies of Southeast Asian tropical cities (which maintained a positive correlation up to a 70% green view index), but aligns with recent Northern European research. The latter found that dense vegetation environments (green view index >75%) may reduce perceived restorativeness, evoking feelings of enclosure and insecurity (Nordh et al., 2009). Domestic eye-tracking studies in university settings have shown that students exhibit dispersed gaze patterns and increased cognitive load indicators within environments with high-density greenery (>65%), providing indirect support for the perceptual saturation hypothesis proposed in this study.

More importantly, whereas the authors of previous vision-based studies suggested an optimal green view index range of 40%–50%, in this study, we found that with the addition of plant aromas, the suitable range expanded to 35%–60%. This validates the “sensory compensation effect”—when the visual channel approaches saturation, positive olfactory input can rebalance one’s overall perception. The highest preference score obtained in this study (*M* = 1.83) significantly exceeds the score (*M* = 1.56) reported in comparable environments using visual stimuli alone, quantitatively demonstrating an approximately 30% improvement of multisensory design over single-sensory design. This finding holds significance for guiding the optimization of landscapes in high-density campus environments.

The High GVI + No Scent condition exhibited the lowest SCL levels but received relatively lower subjective preference ratings (*M* = 5.23, SD = 1.34) compared to medium GVI conditions with olfactory stimuli (*M* = 6.45, SD = 1.12). This physiological-psychological dissociation suggests that the low arousal in high GVI environments may reflect perceptual monotony rather than optimal restoration.

Comparison with GVI Research: International studies on green view index have predominantly assumed linear dose–response relationships. Southeast Asian research found continuous preference increases up to 70% GVI, while North American studies typically recommend 40%–50% optimal ranges. However, these studies examined monocultural visual perception without considering multisensory modulation. The present finding—that olfactory supplementation extends the optimal GVI range from 40%–50% (visual-only) to 35%–60% (multisensory)—reveals a novel compensatory mechanism absent from prior literature. This suggests that current GVI guidelines, derived from visual-only assessments, may underestimate restorative potential in environments where aromatic vegetation is present.

The nonlinear GVI-preference relationship challenges linear assumptions in international research (Wang et al., 2019). Southeast Asian studies report continuous preference increases up to 70% GVI, while Northern European research found dense vegetation (GVI > 75%) reduces restorative ratings by 15% (Jiang et al., 2023). The present finding—that olfactory supplementation extends the optimal GVI range from 40%–50% (visual-only) to 35%–60% (multisensory)—reveals a novel compensatory mechanism absent from prior literature.

### 4.4 Cognitive mechanisms underlying multisensory mitigation of visual saturation

This study makes an important new finding: at high Green View Index (GVI) levels (60%–85%), smell can reduce the negative effects of visual overload. We suggest two ways this may work:

First, attentional load distribution. Our attention has limits. When a scene looks too similar everywhere, it can overwhelm our visual system and cause mental fatigue. Adding a matching scent gives our brain another sensory channel to process. This spreads out the mental effort and reduces strain.

Second, threshold shifting. Recent models show that nature’s benefits follow an upside-down U-shaped curve—they increase up to a point, then decline. Our data indicate that adding smell moves that turning point. In other words, smell lets us tolerate—and even benefit from—more greenery before feeling overloaded.

We also found that rosemary (a herbal scent) worked better than camphor (a woody scent) under high GVI conditions. This may be because rosemary is especially good at boosting alertness, thanks to its main active compound, 1,8-cineole.

### 4.5 Limitations and ecological validity considerations

This study was conducted in a controlled laboratory environment to achieve precise manipulation of visual (GVI levels) and olfactory (plant species) variables while minimizing confounding factors. However, several limitations warrant careful consideration.

First, the use of static two-dimensional photographic stimuli may systematically underestimate two key components of Attention Restoration Theory (ART). The sense of “Extent” is constrained because participants cannot physically navigate or perceive the spatial depth cues inherent to real-world environments. Similarly, the feeling of “Being Away” may be attenuated, as a laboratory setting inherently limits psychological immersion in a natural context. To partially mitigate this, we increased the number of visual scenes and shortened their presentation duration to simulate a more dynamic viewing sequence.

Second, the controlled olfactory presentation protocol—employing a fixed distance (30 cm) and standardized plant materials—differs from the dynamic and variable exposure patterns found in nature. Nevertheless, prior work by Ho et al. (2014), which compared laboratory and field-based olfactory studies, reported comparable physiological responses for short-duration exposures (<10 min), supporting the ecological validity of controlled paradigms for examining acute olfactory effects.

Third, the 25-min core experimental session (within a total procedure of approximately 25–45 min) does not capture the prolonged dwelling or repeated exposure behaviors typical of real campus green space use. Future studies should employ mobile physiological monitoring, ecological momentary assessment, and naturalistic observational designs to validate these findings in authentic behavioral contexts.

## 5 Conclusion

In this study, we address the prominent tendency toward sensory segregation and the insufficient exploration of cross-sensory integration mechanisms in current research on restorative environments on campuses. Adopting a 3 × 3 factorial mixed experimental design, we systematically investigate the interactive effects that the visual green view index and plant-based olfactory stimuli exert on college students’ landscape preferences and restorative experiences in campus green spaces. By integrating multidimensional measurement methods—including eye tracking, physiological monitoring, and psychological assessments—we find that exposure to plant aromas significantly improves participants’ physiological and psychological states, with the rosemary leaf scent demonstrating superior restorative effects compared to the camphor wood scent.

The analysis of the visuo-olfactory interactions revealed a functional differentiation mechanism in environmental perception. Olfactory stimuli were significantly positively correlated with perceived site naturalness, atmosphere, and interestingness, but had weaker correlations with visually dominant dimensions such as plant diversity and color richness. This indicates that the visual system is primarily responsible for spatial structure recognition, while the olfactory system is more involved in emotional regulation and atmosphere creation. The nonlinear interaction between the green view index and olfactory stimuli demonstrated that the medium green view index combined with the leafy scent yielded the highest landscape preference score, whereas the participants evaluated the condition of a high green view index with no scent less favorably. This finding challenges the traditional linear assumption that “a higher green view index is always better.”

The primary contributions of this study include: (1) Methodological innovation through the first controlled factorial experiment examining visual-olfactory integration in campus green spaces; (2) Theoretical advancement by demonstrating nonlinear dose–response relationships and the perceptual saturation phenomenon; (3) Practical implications for multisensory landscape design in high-density educational environments. These findings provide empirical foundations for evidence-based campus planning. Integrating visual greenery design with aromatic vegetation strategically can enhance multisensory restorative experiences.

This study expands the sensory dimensions of Attention Restoration Theory, empirically demonstrating the nonlinear integration characteristics and effects of multisensory environmental perception. The findings reveal that plant aromas directly influence physiological and emotional states and modulate visual landscape evaluations through cross-modal mechanisms, thereby enhancing restorative effects compared to visual-only designs. This discovery offers new insights for optimizing landscapes in high-density campus environments. The authors suggest introducing aromatic plants in areas with moderate green view ratios to strengthen the multisensory synergy while also employing olfactory compensation around buildings with insufficient visual greenery to improve the overall restorative experience. Future research should further explore multisensory integration patterns involving sight, smell, taste, sound, and touch to develop a more comprehensive theoretical framework for restorative environments.

## Data Availability

The original contributions presented in the study are included in the article/supplementary material, further inquiries can be directed to the corresponding author.
